# Notes on Surgical Cases

**Published:** 1860-02

**Authors:** E. Andrews

**Affiliations:** Prof. of Surgery in the Medical Department of Lind University, Surgeon of Mercy Hospital, and Surgeon of Chicago Dispensary


### NOTES ON SURGICAL CASES.

BY E. ANDREWS, M. D.

Prof. of Surgery in the Medical Department of Lind University, Surgeon of Mercy Hospital, and Surgeon of Chicago Dispensary.

*Lithotomy.*—Joseph C., aged 9 years, was admitted to the Surgical wards of Mercy Hospital, on the 10th of December, complaining of incontinence of urine. The penis was small and club shaped, the glans being a little enlarged. He complained of pain in the extremity of the organ, which, however, was not inflamed. The pain was aggravated upon effort at walking or running, and the clothing was wet with urine. I introduced a sound and immediately struck a calculus of considerable size. After a few days preparatory treatment, I brought the patient into the operating room and proceeded to remove the stone in the presence of the students in attendance. The rectum having been previously evacuated, the patient was placed upon the table, chloroform was administered, and the bladder filled with an injection of tepid water. The staff being introduced, I proceeded to perform the lateral operation of lithotomy in the usual manner. The stone, on being extracted proved to be a trifle larger than I had estimated it, being an inch and a half in length, by an inch in breadth. It consisted of alternate layers of uric acid, and phosphates with a uric acid nucleus. A silver tube was inserted in the bladder and the patient replaced in bed. A moderate febrile reaction followed, but at the present time (fourth day after the operation) the symptoms are entirely favorable, and the patient bids fair to have a rapid recovery.

*Necrosis of the Femur.*—Edward Boothe, of Gardner Station, Ill., aged 11 years was admitted to Mercy Hospital with a diseased femur. On examination with the probe, necrosed bone was found in the lower third of the shaft. The knee joint was not affected, but the femur for seven inches above it was much enlarged. Owing to the excellent management of Dr. Rogers, his family physician, the patient was in the enjoyment of a tolerable degree of vigor, notwithstanding the free suppuration which had continued for many months.

I proceeded to remove the sequestrum. A longitudinal incision, five inches in length, was made on the antero-internal aspect of the thigh, down to the bone, crossing one of the sinuses in the track. At the bottom of this sinus a small opening was found leading into a perfect shell of bone, within which the probe detected the sequestrum. I enlarged the opening with the gouge forceps and drew out several fragments of necrosed bone. The cavity in which they lay was about an inch in diameter, four inches long, and cylindrical in form. There were only three very small openings into it. It was obvious that the whole thickness of the lower third of the shaft of the femur had perished down to the epiphysis, and that the periosteum separating from it, had produced this bottle shaped shell of living bone around the sequestrum. Chloroform and aether mixed were administered during the operation. Nothing remarkable occurred during his recovery, and at the present time the incision is rapidly healing.

*Strangulated Hernia.* I was called into the country some twenty miles to see Daniel Wilson, aged 66, who had been lying several days with a strangulated inguinal hernia on the right side. I arrived at midnight, and found the patient as above described. Desirous to accomplish a reduction, if possible, without an operation, I administered chloroform to complete anæsthesia, and resorted to the taxis. The relaxation produced by the chloroform was perfect in the general system, and impressed me very favorably as an agent suitable to be used for this purpose. The structure, however, did not yield, and the attempt was like the previous ones made by Dr. Jones, his family physician, a failure. I proceeded to operate therefore in the usual manner. The sack on being opened contained some clotted blood and serum, and there was considerable fresh lymph gluing the intestines to the sack. There was however no gangrene, and I therefore divided the stricture and returned the gut. There was no serious inflammation and the patient recovered, with a radical cure of the hernia.

*Congenital Hernia Strangulated.*—John H. aged 14, was attacked with a great pain in the groin and scrotum. On examination by his physician, a moderate tumor was found in the scrotum, very hard and elastic, and connected with the external ring by a long and somewhat slender process feeling like a swollen spermatic cord. The patient could give no intelligible account of the history of the case, seeming to be unusually stupid. No testicle could be identified apart from the tumor, so that the physician in attendance was in doubt whether it was a case of hernia or of orchitis. The administration of several brisk cathartics, which only resulted in vomiting and pain, cleared up the matter, and he requested me to meet him and operate. After administering chloroform and ether in equal parts, I made an incision down to the swollen cord. Here I found the intestine stretched in a straight line, from the tumor below to the external ring above, apparently being strangulated at both places, but most firmly below. I divided the upper stricture first, and dissected down to the lower, and divided that also. The gut below the latter point was contained in the sack of the tunica vaginalis, and was a good deal ecchymosed. The strangulation being relieved, the intestine was returned and the parts healed up as usual. There was some local peritonitis, and for several months a good deal of pain on walking, or in any other way moving the body. As this soreness was slow in subsiding, the friends of the patient killed a plump dog, and having extracted his oil they rubbed the patient with it daily. As the soreness afterwards subsided, the dog got more credit for the cure than the surgeon, and the oil of dog is in high repute in that locality to this day.

*Fracture of the Pelvis.*—The patient, a very highly esteemed citizen of V—, Ind., was caught between the side of a freight car and a platform at the station house, in such a way as to compress the lower part of the pelvis, but it was not possible to ascertain the exact direction in which the force was applied.

He was carefully examined and attended by Drs. Cameron and McCarthy, but there was no crepitus, nor other external sign of fracture which the most attentive external examination could detect. The catheter was introduced without difficulty into the bladder, and a pint of pure blood flowed off, but no fragments of bone were touched by the instrument, nor could the finger in the rectum detect any. It was hoped, therefore, that no fatal injury had been done.

A difficulty of expelling the urine supervened, and in a few days a tumor appeared in the hypogastrium like a distended bladder. At the same time, œdema of the perineum and of one thigh took place. As the catheter did not reduce the distension of the bladder in the least, although often introduced, Prof. Davis and myself were called to consult in the case. On my arrival the patient was found delirious, and in a very un promising situation. A careful examination, by introducing the finger into the rectum while the catheter was passed, showed that the instrument made its escape from the urethra at the membranous portion, and passed into the areolar spaces of the pelvis, confirming the suspicions of Dr. Cameron, who had not been able to introduce the instrument in a satisfactory manner after the first or second day from the injury. As no positive sign of fracture could be discovered, I hoped that the rupture of the urethra was the result of a contusion of the soft parts only, and as by no care could the catheter be made to enter the bladder, I determined to relieve that viscus, and also prevent further infiltration of urine by an operation. Chloroform and æther being administered, I introduced a grooved staff and cut down upon it, as in the lateral operation for lithotomy. The staff was found in a cavity from which a gush of urine took place. After this gush, the bladder emptied itself with a free and steady stream, which seemed to come from among the crushed fragments of the prostate gland. On passing my finger along the inner border of the left ischium I found a rough surface from which a large splinter cf bone had evidently been detached; tracing the route of this splinter forward and upward, I at length found it lodged with surprising firmness at the brim of the pelvis, between the bladder and the pubis. It was thin and sharp at the edges, and above two inches and a half in length by an inch or more in breadth. The urethra and prostate gland had been completely destroyed in the path, and other tissues surprisingly cut to pieces, but the coats of the bladder were apparently not pierced. After the operation the patient seemed relieved in a measure of his sufferings, become more rational, and had something like a natural sleep. The urine continued to come away freely by the incision, but there was no permanent improvement and the sufferer died on the following day.

*Fracture of the Pelvis from a fall.*—J. H. a sailor, fell from the mast head of a schooner, and struck the nates upon the deck. I did not see the patient alive, but was present at the *post mortem* examination. Both of the ilia were fractured just external to the sacro-iliac synchondrosis, so that the sacrum was freely movable. The viscera of the pelvis were not wounded, and the sacro-iliac synchondrosis itself remained uninjured, showing the great strength of that articulation. I was not informed of the symptoms preceding the death.

